# Integrative Single‐Cell Transcriptomics, Multi‐Omics Analyses, and Computational Pharmacology Reveal Macrophage Heterogeneity and the Putative THBS1‐CD36 Axis in Coronary Atherosclerosis

**DOI:** 10.1111/cbdd.70399

**Published:** 2026-09-29

**Authors:** Jianpeng Li, Xinrui Liu, Run Shi, Lin Yang, Rong Gu

**Affiliations:** ^1^ Department of Cardiology Nanjing Drum Tower Hospital Clinical College of Nanjing Medical University Nanjing China; ^2^ Department of Cardiology Taizhou Second People's Hospital Affiliated to Yangzhou University Taizhou China; ^3^ Queen Mary College Jiangxi Medical College, Nanchang University Nanchang Jiangxi China; ^4^ Department of Oncology The First Affiliated Hospital of Nanjing Medical University Nanjing China

**Keywords:** coronary atherosclerosis, ligand‐receptor axis, machine learning, macrophage heterogeneity, single‐cell RNA sequencing

## Abstract

Coronary atherosclerosis (CA) is characterized by profound macrophage heterogeneity that drives plaque progression and vulnerability, yet the precise subpopulations and their niche‐specific functions remain incompletely defined. Here, we constructed a single‐cell transcriptomic atlas of human CA using dataset GSE131778 and identified six distinct macrophage subpopulations. Among these, the THBS1+ macrophage subset emerged as a terminally differentiated, hypoxia‐adaptive population with elevated TGF‐β signaling and FOSB‐driven transcriptional regulation. High‐dimensional weighted gene co‐expression network analysis revealed 12 transcriptional modules, with module NEW10 serving as a specific molecular signature of THBS1+ macrophages. Cell–cell communication analysis predicted a THBS1‐CD36 ligand‐receptor interaction potentially contributing to crosstalk between THBS1+ macrophages and lymphatic endothelial cells. Molecular docking and 100‐ns molecular dynamics simulations predicted a thermodynamically favorable and conformationally stable interaction between SMS121 and CD36, providing an initial computational rationale for further experimental evaluation. Finally, leveraging the NEW10 module genes, we trained 13 machine learning classifiers to distinguish ACS from sCAD and evaluated their performance in differentiating ruptured from stable plaques; Support Vector Machine with linear kernel and Naive Bayes achieved the highest accuracy in an independent testing cohort, both attaining an AUC of 0.9333. These findings illuminate macrophage heterogeneity and intercellular communication in CA, identify the predicted THBS1‐CD36 interaction as a candidate for further mechanistic and pharmacological investigation, and establish a macrophage‐derived transcriptional signature for precise risk stratification.

## Graphical abstract

Single‐cell and multi‐omics analyses identified THBS1+ macrophages as a terminally differentiated, hypoxia‐adaptive population driving coronary atherosclerosis through THBS1‐CD36 signaling with lymphatic endothelial cells. Targeting CD36 with SMS121 and leveraging a THBS1+ macrophage gene signature enabled therapeutic prediction and accurate risk stratification.

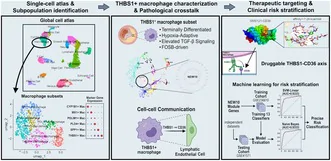

## 1 Introduction

Coronary atherosclerosis (CA) remains the leading cause of morbidity and mortality worldwide, with acute coronary syndrome (ACS) most commonly arising from the rupture or erosion of vulnerable atherosclerotic plaques (Vergallo et al. 2025; Khialani et al. 2025). The pathological transition from stable to unstable plaque is driven by a complex interplay of lipid accumulation, chronic inflammation, and aberrant tissue remodeling within the arterial wall (Pandit and Yurdagul 2025; Qiu et al. 2026). Central to this process are macrophages, which constitute the predominant immune cell population in atherosclerotic lesions and exert multifaceted roles ranging from lipid engulfment and foam cell formation to the secretion of pro‐inflammatory cytokines and matrix‐degrading enzymes (O'Callaghan and Jiang 2025; Teng et al. 2025). Despite their established importance, the full spectrum of macrophage phenotypic diversity and functional specialization within the human coronary plaque microenvironment remains incompletely understood, largely because traditional bulk transcriptomic approaches have obscured cellular heterogeneity (Adachi et al. 2026).

Recent advances in single‐cell RNA sequencing (scRNA‐seq) have substantially improved the characterization of cellular heterogeneity in cardiovascular disease by enabling high‐resolution dissection of cellular ecosystems (Adachi et al. 2026; Ijaz et al. 2026). These technologies have uncovered remarkable heterogeneity among plaque‐resident macrophages, revealing distinct subpopulations with divergent transcriptional programs, including pro‐inflammatory, anti‐inflammatory, resident‐like, and phagocytic states (Adachi et al. 2026). However, the precise classification of macrophage subsets in human CA, their developmental trajectories, underlying gene regulatory networks, and specialized intercellular communication patterns have yet to be fully elucidated (Yoshida et al. 2026; Yang et al. 2025). Furthermore, how specific macrophage subpopulations contribute to plaque destabilization through niche‐specific signaling interactions, and whether their molecular fingerprints can be translated into clinically informative biomarkers, remain important unanswered questions (Xu et al. 2026).

In this study, we constructed a comprehensive single‐cell transcriptomic atlas of human coronary atherosclerotic plaques using dataset GSE131778. Through unsupervised clustering, we identified six transcriptionally distinct macrophage subpopulations, among which the THBS1+ macrophage subset emerged as a functionally unique population characterized by terminal differentiation, hypoxia adaptation, and elevated TGF‐β signaling. Leveraging high‐dimensional weighted gene co‐expression network analysis (hdWGCNA), we delineated 12 transcriptional modules and identified module NEW10 as a specific molecular signature of THBS1+ macrophages. Pseudotemporal trajectory and pySCENIC analyses further identified FOSB as a candidate transcriptional regulator associated with this subset. Cell–cell communication profiling using CellChat predicted a THBS1‐CD36 ligand‐receptor interaction between THBS1+ macrophages and lymphatic endothelial cells, which was not detected in the other analyzed cellular pairings. Computational structural biology analyses, including molecular docking and 100‐ns molecular dynamics simulations, predicted a stable interaction between SMS121 and CD36, providing a preliminary basis for further experimental evaluation. Finally, we used the NEW10 module genes as input features for supervised machine learning and trained 13 classification algorithms to distinguish ACS from sCAD, followed by evaluation of their ability to differentiate ruptured from stable plaques. Our results demonstrated that Support Vector Machine with linear kernel and Naive Bayes showed the highest discriminatory performance among the evaluated models in the independent testing cohort, suggesting that macrophage‐derived transcriptional signatures may have potential value for molecular classification.

Collectively, these findings provide additional insight into macrophage heterogeneity and intercellular crosstalk in coronary plaque pathogenesis, highlight the predicted THBS1‐CD36 interaction for further investigation, and present a single‐cell‐informed machine learning framework for the molecular classification of clinical and morphological manifestations of coronary atherosclerosis.

## 2 Materials and Methods

### 2.1 Single‐Cell RNA Sequencing Data Acquisition and Quality Control

The single‐cell RNA sequencing dataset GSE131778 was downloaded from the Gene Expression Omnibus (GEO) database (Wirka et al. 2019). This dataset comprised coronary plaque tissues from eight human donors. Raw gene expression matrices were processed using the Seurat package (v4.3.0). Initial quality control was performed to filter out low‐quality cells and probable doublets based on the following criteria: cells expressing fewer than 200 genes, genes detected in fewer than three cells, and cells with > 15% mitochondrial‐derived transcripts were excluded. After filtering, a total of 11,756 high‐quality cells were retained for downstream analysis. Raw unique molecular identifier (UMI) counts were normalized using the LogNormalize method with a scale factor of 10,000, followed by natural‐log transformation. The top 2000 highly variable genes were identified using the variance‐stabilizing transformation (vst) method and subsequently scaled for principal component analysis (PCA).

### 2.2 Data Integration, Dimensionality Reduction, and Cell Clustering

Batch effects across the eight individual samples were evaluated and corrected using the Harmony algorithm (Korsunsky et al. 2019) integrated into the Seurat workflow. The first 30 harmonized principal components were selected for dimensionality reduction based on elbow plot and jackStraw analysis. A shared nearest neighbor (SNN) graph was constructed using the FindNeighbors function, followed by graph‐based clustering with FindClusters at a resolution of 0.8. Uniform Manifold Approximation and Projection (UMAP) was applied for two‐dimensional visualization of the cellular landscape. Cell type identities were assigned by mapping cluster‐specific gene expression against established canonical lineage markers for cardiovascular and immune cell types. Differentially expressed genes (DEGs) for each cluster were identified using the Wilcoxon rank‐sum test via the FindAllMarkers function, with thresholds set at |avg_log2FC| > 0.25 and adjusted *p*‐value < 0.05 (Bonferroni correction). Nebula density plots were generated to visualize cluster‐specific cell density distributions. Sankey diagrams illustrating the hierarchical flow from individual patients to samples and subsequently to annotated cell types were constructed using custom plotting pipelines. Cluster‐specific marker expression was visualized using heatmaps and UMAP feature plots.

### 2.3 Macrophage Subset Identification and Re‐Clustering

Macrophages were computationally extracted from the global dataset based on the co‐expression of canonical myeloid lineage markers (e.g., CD68, CD14, CSF1R, CD163) and the absence of markers for other lineages. The resulting 1093 macrophages were subjected to a secondary, independent round of unsupervised clustering using the same Seurat analytical framework with adjusted parameters (resolution = 0.6) to maximize subpopulation resolution. Six transcriptionally distinct macrophage subpopulations were identified and nomenclaturally designated according to their top‐ranking uniquely expressed genes. Proportional abundances were calculated as the percentage of each subtype relative to the total macrophage compartment. Subtype‐specific marker signatures were visualized using dot plots scaled by average expression and percentage of expressing cells.

### 2.4 Gene Ontology Functional Enrichment Analysis

To dissect the functional heterogeneity of macrophage subpopulations, Gene Ontology (GO) enrichment analysis was performed separately for biological process (BP), cellular component (CC), and molecular function (MF) categories using the clusterProfiler package (v4.6.0) (Yu et al. 2012). The background gene set comprised all genes detected in the macrophage subset. Enrichment significance was determined using the hypergeometric test with Benjamini‐Hochberg false discovery rate (FDR) correction. GO terms with an adjusted *p*‐value < 0.05 were considered statistically significant and were visualized using dot plots ranked by gene ratio and enrichment significance.

### 2.5 High‐Dimensional Weighted Gene Co‐Expression Network Analysis (hdWGCNA)

The hdWGCNA pipeline was applied to the macrophage subset to construct a single‐cell weighted gene co‐expression network (Morabito et al. 2023). A soft‐thresholding power (*β*) was selected by evaluating the scale‐free topology fitting index and mean connectivity across a range of powers (1–20); a power value of 8 was chosen as the lowest power achieving the predefined scale‐free topology criterion, with *R* ^2^ = 0.8044 and a mean connectivity of 67.96. Adjacency matrices were transformed into topological overlap matrices (TOMs), and hierarchical clustering was performed using 1‐TOM as the distance metric. Modules were identified via the dynamic tree cut algorithm (deepSplit = 2, minModuleSize = 50), and modules with eigengene correlations greater than 0.75 were merged using cutHeight = 0.25 to reduce redundancy among modules with highly similar expression patterns. Module eigengenes (MEs) were calculated as the first principal component of each module's gene expression matrix. Module‐trait relationships were assessed by correlating MEs with macrophage subtype identities. Hub genes were defined as those exhibiting high module membership (kME > 0.7) and high gene significance for subtype identity. Module activity scores were projected onto the macrophage UMAP embedding using the ModuleUMAP function. Inter‐module correlation matrices and hub gene co‐expression networks were visualized using the igraph package.

### 2.6 Pseudotemporal Trajectory Reconstruction

To infer developmental relationships among macrophage subtypes, we employed Monocle 2 (Qiu et al. 2017). The ordering genes were selected based on differential expression among the six macrophage subpopulations (*q*‐value < 0.01). Dimensionality reduction was performed using the Discriminative Dimensionality Reduction with Trees (DDRTree) algorithm, and cells were ordered along the inferred pseudotime trajectory. Branch points representing putative cell‐fate decisions were identified algorithmically. Differential expression testing along pseudotime was conducted using the differential GeneTest function, and genes with significant pseudotime‐dependent expression (*q*‐value < 0.01) were clustered into temporal expression profiles. Smoothed expression curves for representative subtype markers were fitted using generalized additive models (GAMs) and plotted as a function of pseudotime.

### 2.7 Transcription Factor Regulon Inference and Pathway Scoring

Transcription factor (TF) regulon activity was inferred using the pySCENIC pipeline (Aibar et al. 2017). Briefly, gene regulatory networks were reconstructed using the GRNBoost2 algorithm, followed by cis‐regulatory motif enrichment analysis using cisTarget databases to prune indirect targets and retain only direct TF‐target relationships (regulons). Regulon activity was scored per cell using the AUCell algorithm, yielding area under the recovery curve (AUC) values that were normalized across the dataset. Average regulon activity and differential activation across macrophage subtypes were visualized using heatmaps and violin plots. Gene set scoring for hypoxia and TGF‐β signaling pathways was performed using the AddModuleScore function in Seurat, employing curated gene signatures derived from the Molecular Signatures Database (MSigDB, v7.5) and literature‐curated gene sets (Liberzon et al. 2015).

### 2.8 Intercellular Communication Analysis

Cell–cell communication networks were inferred using the CellChat algorithm with its integrated database of ligand‐receptor interactions (Jin et al. 2025, 2021). The analysis was conducted on the complete cellular atlas, with a focus on outgoing and incoming signaling patterns of macrophage subpopulations. Communication probabilities were computed using a truncated mean expression model with empirical priors. Signaling pathways of interest were extracted and visualized as network circle plots, heatmaps of sender‐receiver interaction probabilities, and bubble plots depicting ligand‐receptor pair contributions. The specific THBS1‐CD36 ligand‐receptor axis was manually inspected and highlighted to identify subtype‐specific communication channels.

### 2.9 Protein Structure Preparation, Molecular Docking, and Interaction Analysis

The three‐dimensional structural coordinates of human CD36 and THBS1 were retrieved from the Protein Data Bank (PDB) or predicted using AlphaFold2 where experimental structures were unavailable. SMS121 [2‐[4‐[(E)‐3‐[4‐(dimethylamino)phenyl]‐3‐oxoprop‐1‐enyl]‐2‐methoxyphenoxy]acetic acid] was originally identified through receptor‐based virtual screening of an approximately 32,000‐compound subset of the Enamine database and subsequently characterized as a small‐molecule CD36 inhibitor (Åbacka et al. 2024). Previous surface plasmon resonance analysis reported a CD36‐binding affinity of approximately 5 μM, while cellular assays showed inhibition of fatty‐acid uptake in acute myeloid leukemia cells with an IC_50_ of 164 μM (Åbacka et al. 2024). The small‐molecule compound SMS121 [PubChem CID: 2539449] was retrieved from a chemical database and prepared for docking by energy minimization, rotatable bond assignment, and generation of low‐energy 3D conformers using AutoDock Tools. Molecular docking of SMS121 into the predicted ligand‐binding pocket of CD36 was performed using AutoDock Vina (v1.1.2). A search space grid box encompassing the entire binding cavity was defined with a grid spacing of 0.375 Å. The docking pose exhibiting the most favorable binding free energy (affinity = −7.24 kcal/mol) was selected as the optimal conformation. Two‐dimensional interaction diagrams were generated using LigPlot+ (v2.2) to visualize hydrogen bonds, hydrophobic contacts, and van der Waals interactions between SMS121 and surrounding amino acid residues within the CD36 binding pocket.

### 2.10 Molecular Dynamics Simulations and Free Energy Profiling

Molecular dynamics (MD) simulations of the SMS121‐CD36 complex were conducted using GROMACS (v2022.3) with the AMBER99SB‐ILDN force field for the protein and the General AMBER Force Field (GAFF2) for the ligand, parameterized using the Antechamber suite with AM1‐BCC partial charges. The complex was solvated in a cubic TIP3P water box extending 10 Å beyond the protein surface, and the system was neutralized by adding Na^+^ and Cl^−^ counterions. Energy minimization was performed using the steepest descent algorithm (50,000 steps), followed by equilibration under NVT (1 ns) and NPT (1 ns) ensembles with position restraints on the protein heavy atoms. A production MD run of 100 ns was executed under periodic boundary conditions at 300 K and 1 bar, maintained using the V‐rescale thermostat and Parrinello‐Rahman barostat. Trajectory snapshots were saved every 10 ps for subsequent analysis. Conformational stability was assessed by calculating the root‐mean‐square deviation (RMSD) of the protein backbone, ligand heavy atoms, and the overall complex, as well as the radius of gyration (Rg), total number of hydrogen bonds, and solvent‐accessible surface area (SASA) using GROMACS built‐in analysis tools. Gibbs free energy landscapes were constructed from the MD trajectories by projecting the probability distributions onto RMSD and Rg reaction coordinates using the gmx sham module, with energy values expressed in kJ/mol.

### 2.11 Machine Learning Classification and Model Evaluation

Among the 12 hdWGCNA‐derived modules, NEW10 was selected because its genes showed preferential expression in THBS1+ macrophages compared with the other macrophage subpopulations. To develop a transcriptional signature associated with coronary atherosclerotic instability, the hub genes from the hdWGCNA‐derived NEW10 module (characteristic of THBS1+ macrophages) were used as the input feature set. The training cohort comprised dataset GSE236610, consisting of 14 acute coronary syndrome (ACS) and 13 stable coronary artery disease (sCAD) samples which were classified according to clinical presentation (Widlansky et al. 2023). The independent testing cohort comprised dataset GSE41571, consisting of 5 ruptured plaque and 6 stable plaque samples, which were classified according to plaque morphology (Lee et al. 2013). Because ACS versus sCAD and ruptured versus stable plaques represent related but non‐equivalent dimensions of coronary atherosclerotic instability, the independent testing cohort was used to assess whether the ACS‐associated transcriptional signature could generalize to morphological plaque instability.

Thirteen supervised binary classification algorithms were implemented in Python (v3.9) using the scikit‐learn (v1.2.0) library: Support Vector Machine with linear kernel (SVM‐Linear), Support Vector Machine with radial basis function kernel (SVM‐RBF), Gaussian Naive Bayes, Multi‐Layer Perceptron (Neural Network), Elastic Net logistic regression, Random Forest, XGBoost, Lasso logistic regression, Ridge logistic regression, standard Logistic Regression, Linear Discriminant Analysis (LDA), Decision Tree, and K‐Nearest Neighbors (KNN, *k* = 5). Features were standardized using *Z*‐score normalization prior to model training. Hyperparameter optimization was performed for each algorithm using 5‐fold cross‐validation with grid search on the training set. Model discriminatory performance on the independent testing set was quantified using the area under the receiver operating characteristic curve (AUC), sensitivity, and specificity. ROC curves were plotted for the top‐performing classifiers. Model‐derived classification scores from the SVM‐Linear and Naive Bayes models were compared between ruptured and stable plaque groups using appropriate statistical tests.

### 2.12 Statistical Analysis

All statistical analyses were performed in R or Python. Differences in continuous variables between two groups were assessed using the two‐sided Wilcoxon rank‐sum test (Mann–Whitney *U* test) or Student's *t*‐test, depending on the normality of distribution as evaluated by the Shapiro–Wilk test. Comparisons of model‐derived classification scores between ruptured and stable plaque groups were performed using the two‐sided Wilcoxon rank‐sum test. Categorical variables were analyzed using Fisher's exact test or chi‐squared test where applicable. Correlation analyses were performed using Pearson or Spearman methods. For multiple comparisons, *p*‐values were adjusted using the Benjamini‐Hochberg method. A two‐sided *p*‐value < 0.05 was considered statistically significant unless otherwise specified. Data visualization was performed using ggplot2, ComplexHeatmap, and custom plotting functions in R software.

## 3 Results

### 3.1 Single‐Cell Transcriptomic Landscape of Human Coronary Atherosclerosis

To systematically characterize the cellular heterogeneity in coronary atherosclerosis (CA), we analyzed the single‐cell RNA sequencing dataset GSE131778, which comprised a total of 11,756 high‐quality cells from eight human samples. Unsupervised clustering and Uniform Manifold Approximation and Projection (UMAP) visualization revealed 18 distinct cell populations, including T cells, NK cells, B cells, plasma cells, monocytes, cycling myeloid cells, macrophages, dendritic cells, mast cells, neutrophils, epithelial cells, fibroblasts, smooth muscle cells, pericytes, Schwann cells, lymphatic endothelial cells (LECs), arterial endothelial cells, and venous endothelial cells (Figure 1A). The nebula plot further illustrated the density distribution of these cellular clusters, showing the separation and relative distribution of the annotated populations (Figure 1B). Sankey diagram tracing illustrated the distribution of the major cell types across individual patients and samples (Figure 1C). A comprehensive heatmap of canonical marker genes validated the identity of each cluster, showing high and selective expression of established lineage‐specific markers (Figure 1D). Furthermore, feature plots overlaid on the UMAP embedding confirmed the spatial expression patterns of representative marker genes for each cell type, providing additional support for the cell‐type annotations (Figure 1E). Collectively, these data provide a single‐cell overview of the CA microenvironment and a basis for subsequent analyses of cellular heterogeneity.

**FIGURE 1:**
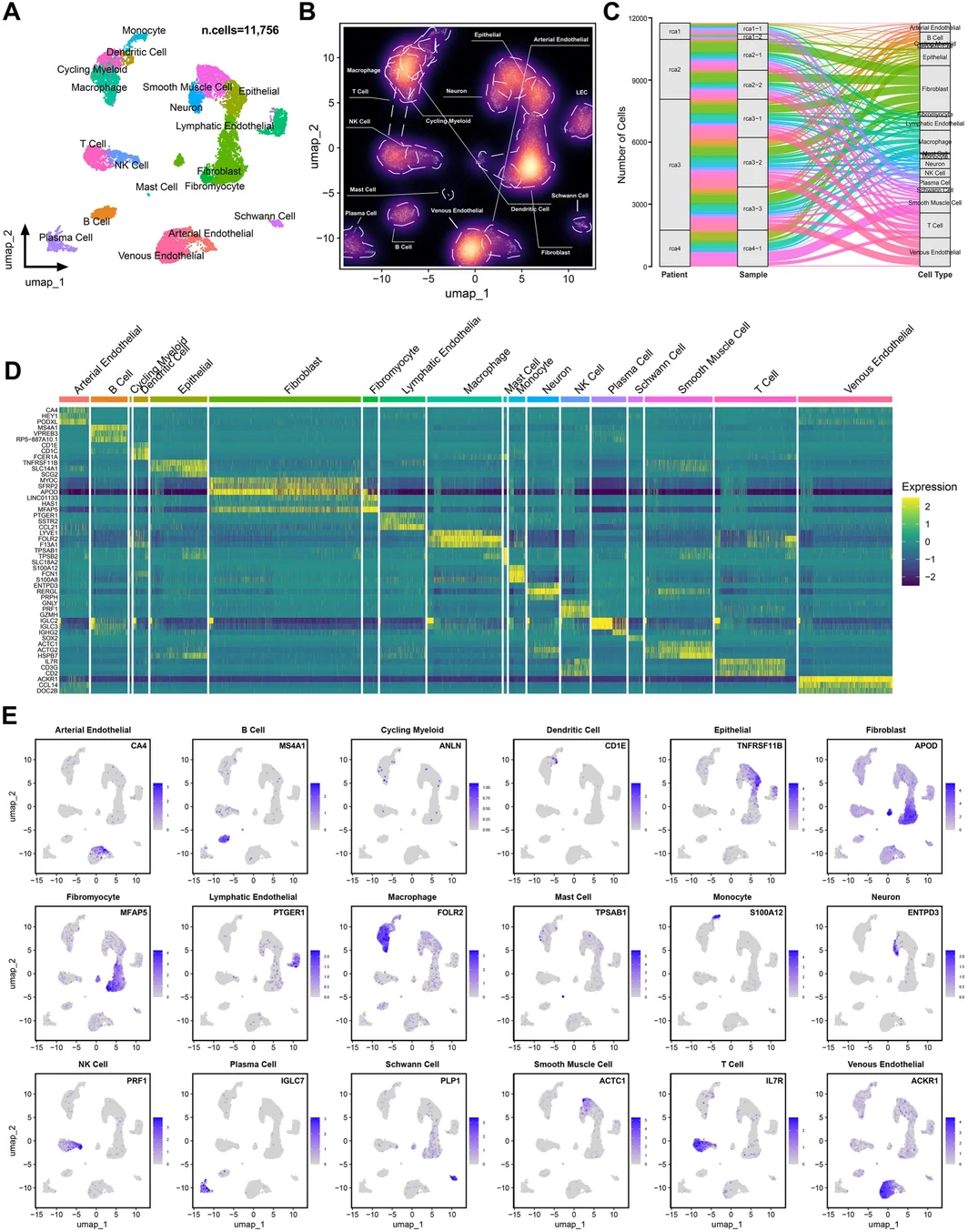
Single‐cell transcriptomic atlas of human coronary atherosclerosis. (A) UMAP projection of 11,756 cells from human coronary atherosclerosis, color‐coded by 18 identified cell types. (B) Density plot showing the distribution of cells across the UMAP embedding. (C) Sankey diagram illustrating the cell distribution across individual patients, samples, and annotated cell types. (D) Heatmap displaying the expression levels of canonical marker genes across the defined cell clusters. (E) UMAP feature plots depicting the expression of representative marker genes for each cell type.

### 3.2 Heterogeneous Subpopulations of Macrophages Exhibit Distinct Transcriptional Identities in Coronary Atherosclerosis

Given the central role of macrophages in atherosclerotic plaque development and instability, we next extracted 1093 macrophages for re‐clustering analysis. This revealed six transcriptionally distinct macrophage subpopulations, designated as CYP1B1+ Macrophage (*n* = 207), MX1+ Macrophage (*n* = 96), PDLIM1+ Macrophage (*n* = 75), PLD4+ Macrophage (*n* = 284), SPP1+ Macrophage (*n* = 206), and THBS1+ Macrophage (*n* = 225) (Figure 2A). Proportional analysis showed that PLD4+ macrophages constituted the largest fraction (26.0%), followed by THBS1+ (20.6%), CYP1B1+ (18.9%), SPP1+ (18.8%), MX1+ (8.8%), and PDLIM1+ (6.9%) macrophages (Figure 2B). Dot plot visualization of subtype‐specific marker genes demonstrated unique transcriptional signatures for each subpopulation, with minimal overlap in their defining gene sets (Figure 2C). To gain functional insights, we performed Gene Ontology (GO) enrichment analyses across biological process (BP), cellular component (CC), and molecular function (MF) categories. The GO‐BP analysis revealed that individual subpopulations were enriched in distinct immunological and metabolic processes, such as antigen processing and presentation, inflammatory responses, and lipid metabolism (Figure 2D). GO‐CC analysis indicated differential compartmentalization, with specific subgroups showing enrichment in endosomal, lysosomal, or extracellular matrix‐associated components (Figure 2E). Meanwhile, GO‐MF analysis highlighted divergent molecular activities, including cytokine binding, receptor‐ligand interactions, and enzymatic functions (Figure 2F). These findings demonstrate functional heterogeneity among macrophage subpopulations within the CA plaque microenvironment and suggest potential differences in their biological roles.

**FIGURE 2:**
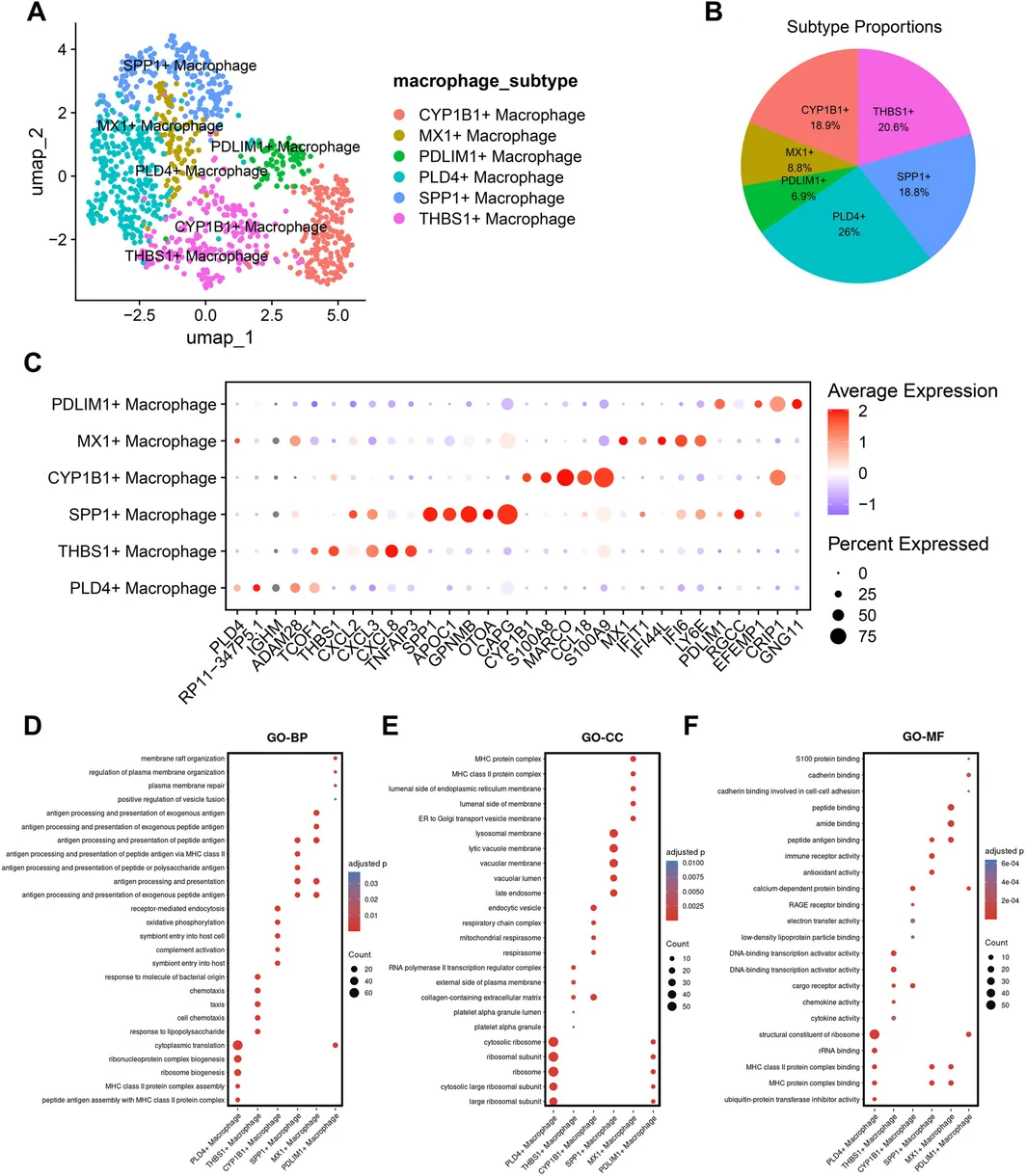
Heterogeneous subpopulations of macrophages exhibit distinct transcriptional identities in coronary atherosclerosis. (A) UMAP plot of 1093 re‐clustered macrophages, color‐coded by six identified subpopulations: CYP1B1+, MX1+, PDLIM1+, PLD4+, SPP1+, and THBS1+ macrophages. (B) Pie chart showing the proportional distribution of the six macrophage subtypes. (C) Dot plot displaying the expression of subtype‐specific marker genes across the subpopulations. (D) Dot plot illustrating Gene Ontology (GO) biological process enrichment for each subtype. (E) Dot plot illustrating GO cellular component enrichment for each subtype. (F) Dot plot illustrating GO molecular function enrichment for each subtype.

### 3.3 High‐Dimensional Weighted Gene Co‐Expression Network Analysis Reveals Modular Transcriptional Programs Underlying Macrophage Diversity

To dissect the gene regulatory architecture driving macrophage heterogeneity, we applied high‐dimensional weighted gene co‐expression network analysis (hdWGCNA). Scale‐free topology fitting and connectivity analyses supported the selection of a soft‐thresholding power of 8, which yielded a scale‐free topology fitting index of *R* ^2^ = 0.8044 and a mean connectivity of 67.96 (Figure 3A). Hierarchical clustering of the gene co‐expression dendrogram delineated 12 distinct transcriptional modules, labeled NEW1 through NEW12 and represented by unique color codes (Figure 3B). Each module was characterized by its top 10 hub genes, which exhibited strong intra‐module connectivity and were considered representative of the module's transcriptional program (Figure 3C). UMAP‐based visualization of module activity scores across all macrophage subpopulations revealed spatially restricted patterns, indicating that specific modules were preferentially activated in discrete macrophage subsets (Figure 3D). Correlation analysis among the 12 modules demonstrated positive and negative inter‐module associations (Figure 3E). Dot plot analysis further quantified the average expression and percentage of cells expressing module‐enriched genes within each macrophage subtype; notably, black‐bordered boxes highlighted modules that were preferentially enriched in specific subpopulations, revealing a tight coupling between transcriptional modules and cellular phenotypes (Figure 3F). Network visualization of hub genes across all 12 modules illustrated their co‐expression patterns and interconnectivity among macrophage subgroups (Figure 3G). Finally, subpopulation‐specific module networks provided a granular view of how individual macrophage subtypes were governed by unique gene regulatory circuits (Figure 3H). Together, these results identify transcriptional modules associated with distinct macrophage subpopulation identities in CA.

**FIGURE 3:**
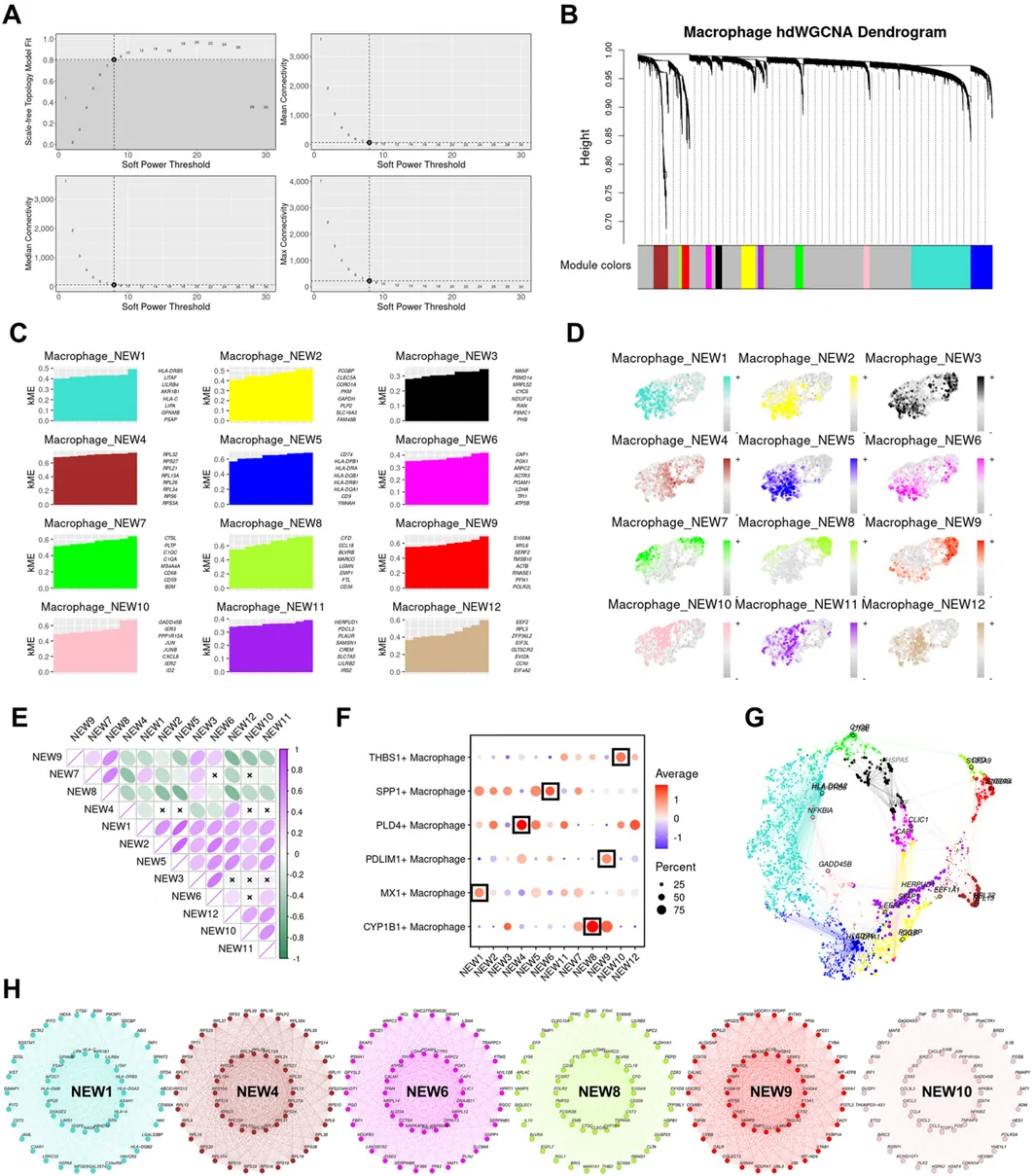
High‐dimensional weighted gene co‐expression network analysis reveals modular transcriptional programs underlying macrophage diversity. (A) Scale‐free topology fitting and mean connectivity analyses for soft‐thresholding power selection. (B) Hierarchical clustering dendrogram of genes, with module assignments indicated by the color bar below. (C) Module eigengene (ME) values and top 10 hub genes for each of the 12 identified modules. (D) UMAP visualization of module activity scores across macrophage subpopulations. (E) Correlation matrix of the 12 gene modules. (F) Dot plot displaying the average expression and percentage of cells expressing module‐enriched genes across macrophage subtypes, with black borders marking highlighted associations. (G) Network visualization of hub genes across all modules. (H) Network diagrams for the selected subpopulation‐specific modules.

### 3.4 Pseudotemporal Trajectory and Transcription Factor Regulon Dynamics Reveal Divergent Differentiation Programs Among Macrophage Subtypes

To infer the developmental relationships among macrophage subpopulations, we employed Monocle 2 to reconstruct pseudotemporal trajectories. The branched trajectory structure, color‐coded by the six macrophage subtypes, revealed a non‐linear differentiation landscape with distinct branch points (Figure 4A). Pseudotime progression, represented by a gradient from dark to light coloring, delineated the transition from early to terminal positions along the inferred trajectory (Figure 4B). State assignment analysis partitioned cells into distinct trajectory states, further supporting the presence of divergent differentiation fates (Figure 4C). Heatmap visualization of differentially expressed genes along pseudotime, coupled with Gene Ontology Biological Process (GOBP) enrichment analysis of gene clusters, identified temporally regulated transcriptional programs, including immune activation, lipid handling, and apoptotic processes (Figure 4D). Fitted expression curves of representative marker genes for each subtype confirmed their dynamic regulation across pseudotime, with several genes exhibiting either gradual upregulation or downregulation as cells progressed toward terminal states (Figure 4E). Notably, gene set scoring revealed that THBS1+ macrophages displayed the highest hypoxia signature among all subpopulations (Figure 4F), as well as the strongest TGF‐β signaling activity (Figure 4G), consistent with a hypoxia‐responsive and TGF‐β‐enriched transcriptional state.

**FIGURE 4:**
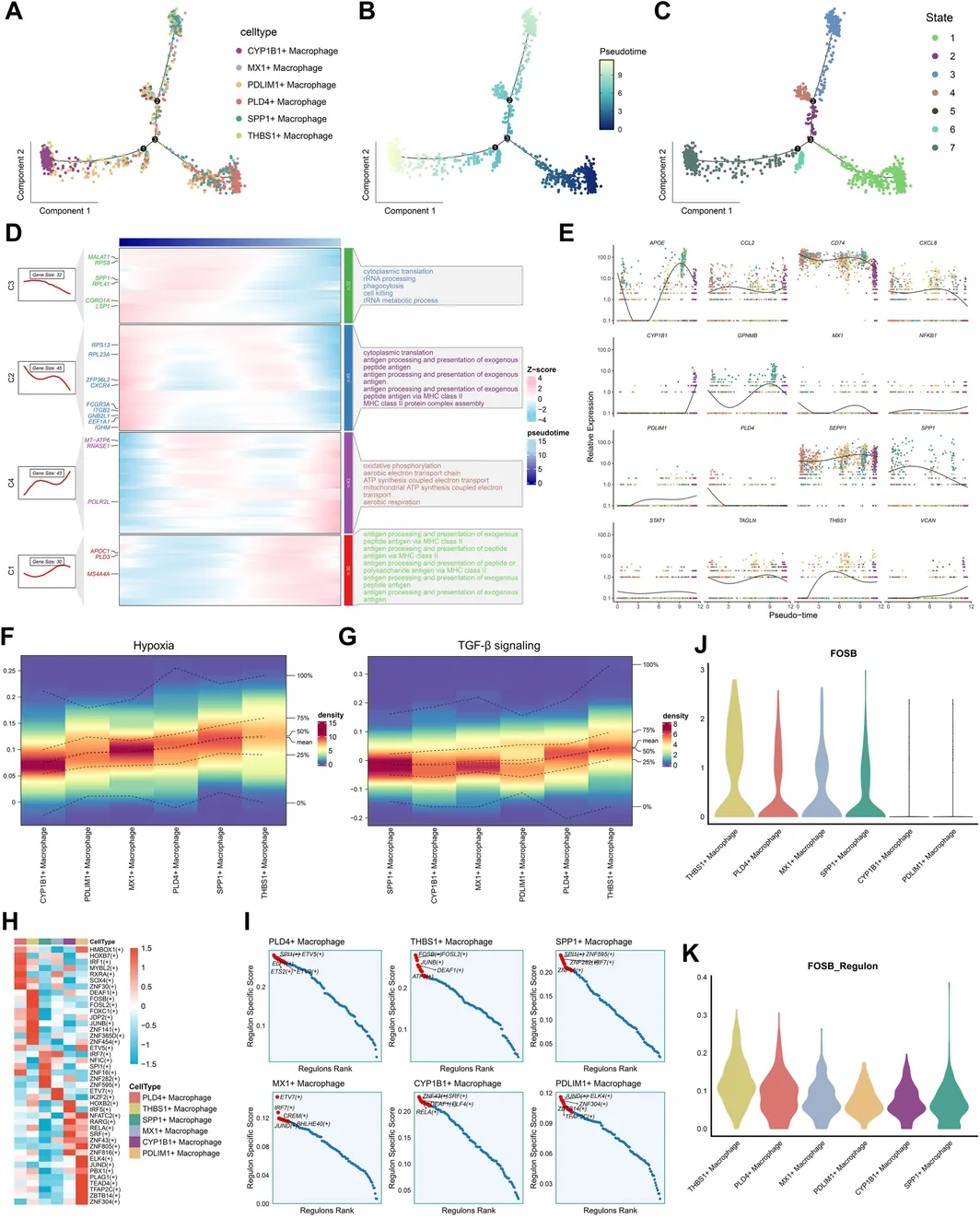
Pseudotemporal trajectory and transcription factor regulon dynamics reveal divergent differentiation programs among macrophage subtypes. (A) Monocle 2 pseudotemporal trajectory of macrophage subpopulations, color‐coded by the six subtypes. (B) Trajectory plot color‐coded by pseudotime progression. (C) Trajectory plot showing the assignment of cells to distinct trajectory states. (D) Heatmap of differentially expressed genes along pseudotime, with annotations for GO biological process enrichment. (E) Smoothed expression curves of subtype‐representative marker genes along pseudotime. (F) Gene set scoring plot illustrating hypoxia signature activity across subtypes. (G) Gene set scoring plot illustrating TGF‐β signaling activity across subtypes. (H) Heatmap of average transcription factor (TF) regulon activity across macrophage subtypes. (I) Ranking plots showing top specifically activated TF regulons for each subtype. (J) Violin plots showing FOSB expression across subtypes. (K) Violin plots showing FOSB regulon activity across subtypes.

To investigate the transcriptional regulatory mechanisms governing these phenotypes, we performed pySCENIC analysis to infer transcription factor (TF) regulon activity. The heatmap of average TF activity across subtypes demonstrated distinct regulon activation patterns, with each macrophage subset characterized by a unique combination of active TFs (Figure 4H). Ranking analysis by regulon‐specific scores identified the top five subtype‐enriched candidate TFs in each subpopulation (Figure 4I). Among these, FOSB emerged as a particularly notable candidate: violin plots showed that FOSB expression was highest in THBS1+ macrophages compared to all other subtypes (Figure 4J), and correspondingly, FOSB regulon activity was markedly elevated in this population (Figure 4K). These integrated trajectory and regulon analyses suggest that THBS1+ macrophages occupy a terminal position along the inferred trajectory and exhibit a hypoxia‐responsive transcriptional state associated with elevated FOSB regulon activity.

### 3.5 Cell–Cell Communication Profiling Identifies THBS1+ Macrophage‐Mediated Signaling Rewiring in the Coronary Atherosclerotic Microenvironment

We next interrogated how macrophage subpopulations interact with the broader cellular ecosystem using CellChat. Network visualization of intercellular communication revealed that each macrophage subtype was predicted to establish distinct ligand‐receptor connections with neighboring cell types, including endothelial cells, smooth muscle cells, fibroblasts, and lymphocytes (Figure 5A). Lollipop plots quantifying interaction counts between all cell populations and THBS1+ macrophages indicated that this subpopulation was predicted to engage in extensive crosstalk with multiple cellular compartments (Figure 5B). Pathway‐level analysis revealed reduced predicted MIF signaling in THBS1+ macrophages compared with other subtypes (Figure 5C), whereas predicted THBS signaling was markedly enhanced in this population (Figure 5D). Heatmaps of sender‐receiver communication probabilities for the MIF and THBS pathways illustrated the directional flow of these predicted signals across the cellular landscape, with THBS1+ macrophages serving as prominent senders in the THBS pathway but diminished contributors to MIF‐mediated communication (Figure 5E,F). Ligand‐receptor interaction analysis predicted a THBS1‐CD36 interaction between THBS1+ macrophages and lymphatic endothelial cells (LECs) that was not detected for the other macrophage subpopulations (Figure 5G). These data indicate that THBS1+ macrophages exhibit a distinct predicted communication profile characterized by reduced MIF signaling and enhanced THBS‐mediated interactions. The functional consequences of these predicted interactions require experimental validation.

**FIGURE 5:**
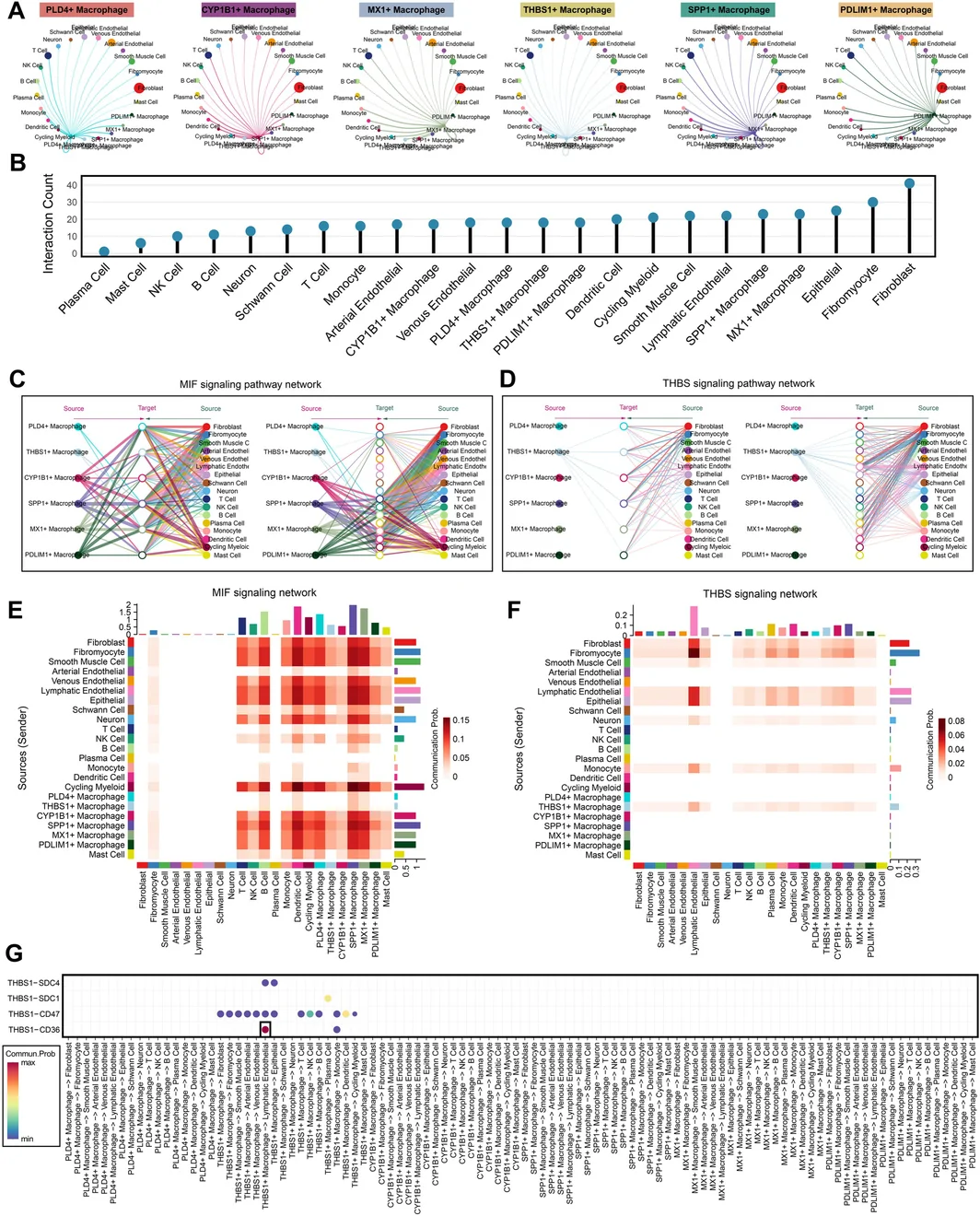
Cell–cell communication profiling identifies THBS1+ macrophage‐mediated signaling rewiring in the coronary atherosclerotic microenvironment. (A) Network visualization showing the intercellular connections between each macrophage subtype and other cell types. (B) Lollipop plot showing the cell–cell interaction counts across the different cell populations. (C) Network diagram illustrating the MIF signaling pathway interactions. (D) Network diagram illustrating the THBS signaling pathway interactions. (E) Heatmap showing the communication probabilities within the MIF signaling network. (F) Heatmap showing the communication probabilities within the THBS signaling network. (G) Ligand‐receptor interaction plot displaying the THBS1‐CD36 interactions.

### 3.6 Molecular Docking and Molecular Dynamics Simulations Demonstrate High‐Affinity Binding of SMS121 to the CD36 Receptor

Given the identification of the THBS1‐CD36 axis as a specific communication channel between THBS1+ macrophages and lymphatic endothelial cells (LECs), we explored the therapeutic potential of targeting this interaction. Schematic modeling illustrated the proposed mechanism whereby THBS1+ macrophages might signal to lymphatic endothelial cells (LECs) via THBS1 binding to CD36 (Figure 6A). Protein–protein docking simulations visualized the global spatial conformation of the THBS1‐CD36 complex, revealing complementary surface interactions (Figure 6B,C). We then investigated whether the small‐molecule compound SMS121 could disrupt this axis by binding directly to CD36. Molecular docking analysis demonstrated that SMS121 occupied the ligand‐binding pocket of CD36 with favorable spatial complementarity (Figure 6D). Detailed interaction analysis revealed a binding affinity of −7.24 kcal/mol, suggesting favorable predicted binding within the applied docking model (Figure 6E). The two‐dimensional interaction map further delineated the binding mode, showing hydrogen bonds, van der Waals forces, and hydrophobic interactions between SMS121 and key amino acid residues within the CD36 active pocket (Figure 6F). To validate the stability of this interaction, we conducted 100‐ns molecular dynamics simulations. Time‐course trajectories of root‐mean‐square deviation (RMSD) for the ligand, complex, and protein, together with radius of gyration, hydrogen bond counts, and solvent‐accessible surface area, all converged to stable equilibria, confirming the robustness of the SMS121‐CD36 complex (Figure 6G). Two‐dimensional and three‐dimensional Gibbs free energy landscapes revealed a well‐defined low‐energy binding basin, further supporting the thermodynamic favorability and conformational stability of the SMS121‐CD36 interaction (Figure 6H,I). These computational findings provide preliminary structural support for the predicted interaction between SMS121 and CD36 and warrant further experimental evaluation of its binding and potential effects on THBS1‐CD36 signaling in CA.

**FIGURE 6:**
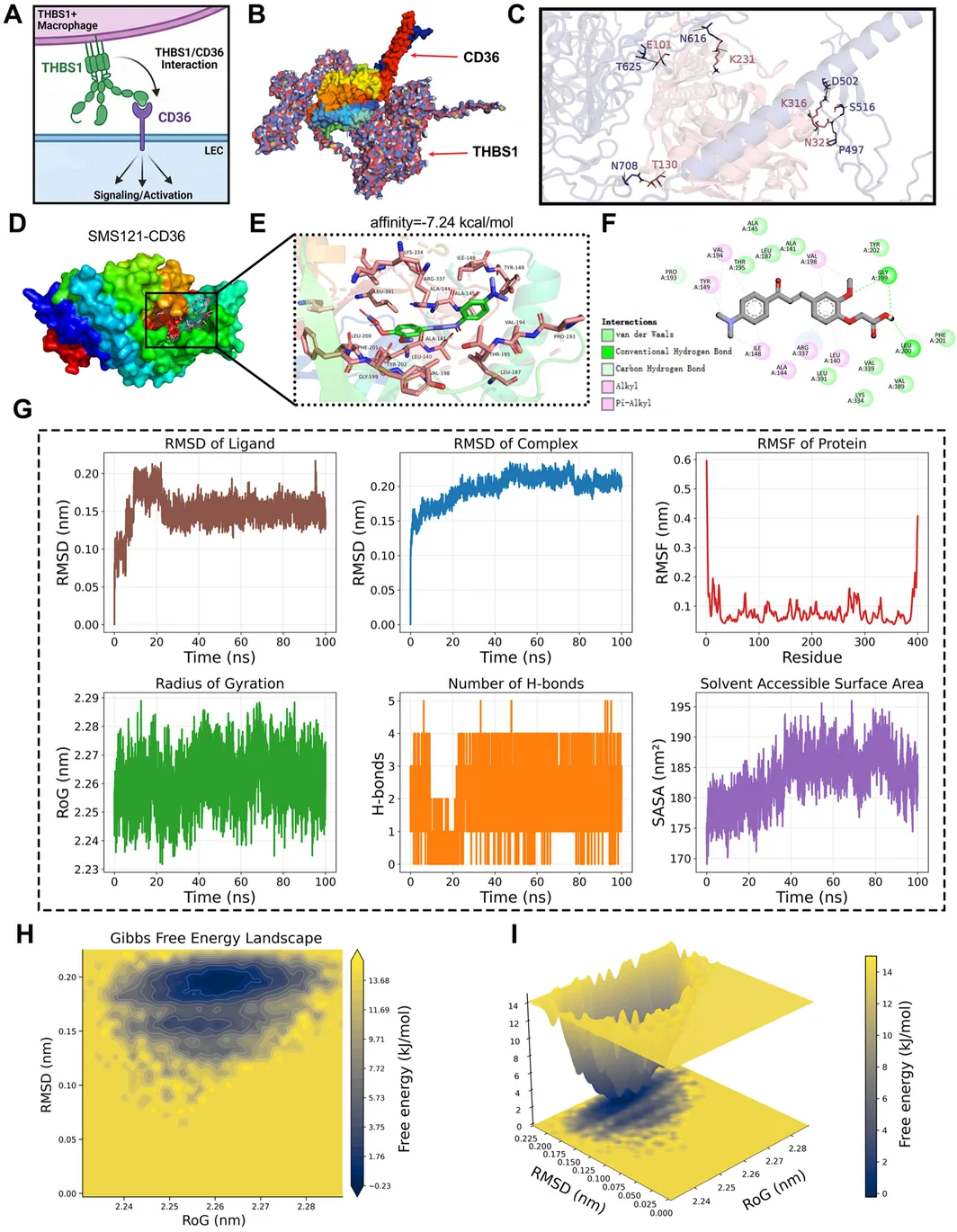
Molecular docking and dynamics simulations demonstrate high‐affinity binding of SMS121 to the CD36 receptor. (A) Schematic diagram illustrating the THBS1‐CD36 signaling axis. (B) 3D structural model of the THBS1‐CD36 complex. (C) Magnified view of the THBS1‐CD36 binding interface. (D) Molecular docking model of SMS121 within the CD36 binding pocket. (E) Binding interaction map displaying the calculated affinity score. (F) 2D interaction map showing the interactions between SMS121 and CD36 amino acid residues. (G) Time‐course trajectories from molecular dynamics simulations showing RMSD, radius of gyration, hydrogen bond counts, and solvent‐accessible surface area. (H) 2D Gibbs free energy landscape. (I) 3D Gibbs free energy landscape.

### 3.7 Machine Learning Evaluation of the THBS1+ Macrophage Gene Signature Across Clinical and Morphological Manifestations of Coronary Atherosclerosis

To evaluate the translational potential of the THBS1+ macrophage‐associated signature, we leveraged the hdWGCNA‐derived NEW10 module, which represents the transcriptional signature of THBS1+ macrophages, as a feature set for machine learning‐based classification. We trained 13 distinct binary classifiers using the GSE236610 dataset (14 acute coronary syndrome [ACS] and 13 stable coronary artery disease [sCAD] samples) and evaluated their performance on the independent testing set GSE41571 (5 ruptured plaque and 6 stable plaque samples). The models were trained to distinguish ACS from sCAD and were subsequently tested for their ability to distinguish ruptured from stable plaques. These two classification tasks reflect related but non‐equivalent aspects of coronary atherosclerotic instability.

Bar plot comparison of area under the receiver operating characteristic curve (AUC) values revealed substantial variability across algorithms (Figure 7A). Support Vector Machine with linear kernel (SVM Linear) and Naive Bayes achieved the highest discriminatory performance for ruptured versus stable plaques in the independent testing cohort, both attaining an AUC of 0.9333, followed by Neural Network (AUC = 0.8667). In contrast, KNN (AUC = 0.4333), SVM RBF, LDA, and Decision Tree (all AUC = 0.5) showed limited ability to distinguish ruptured from stable plaques (Figure 7A). The ROC curves for SVM Linear and Naive Bayes were consistent with their corresponding AUC values (Figure 7B,C).

**FIGURE 7:**
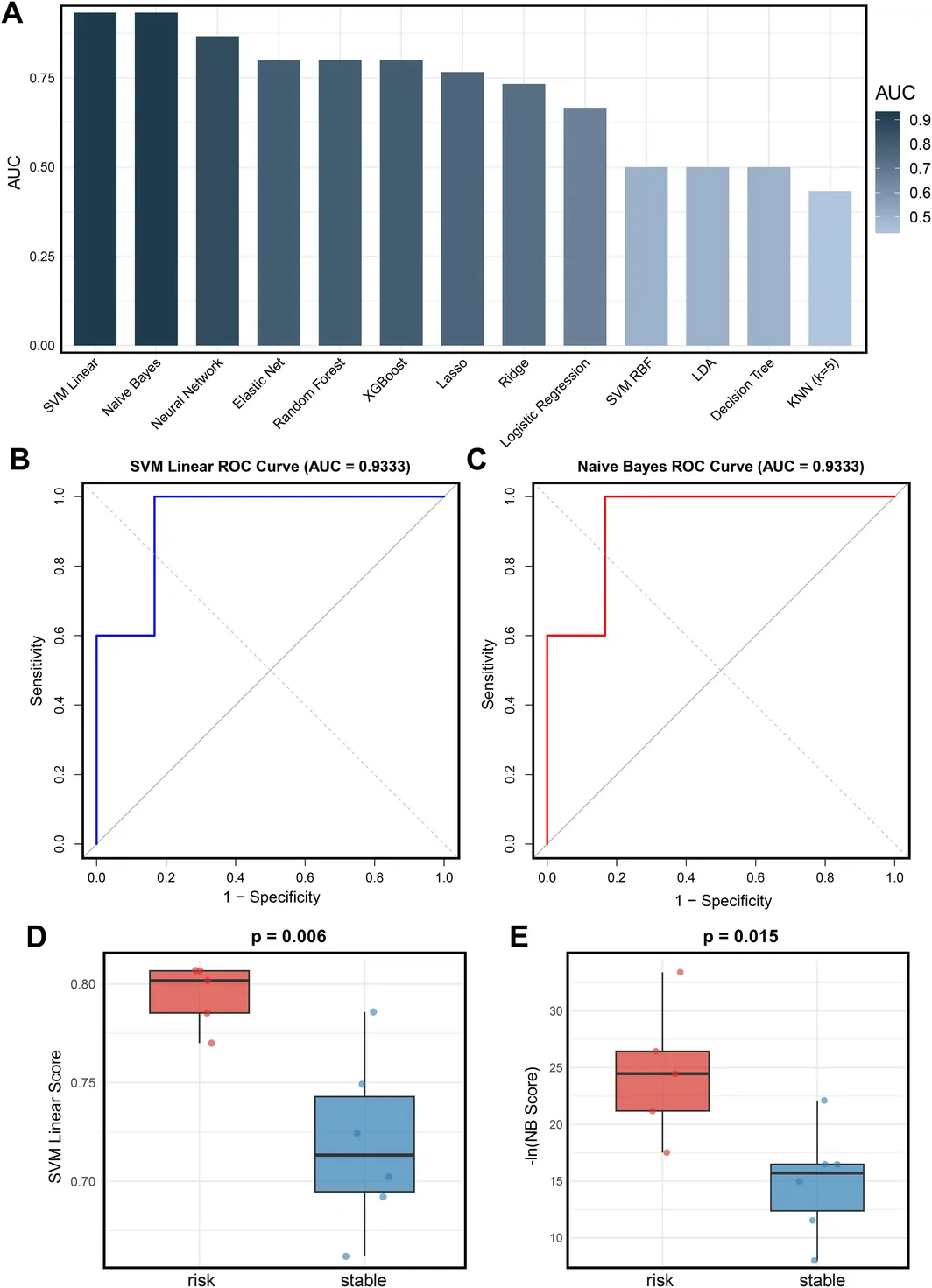
Machine learning classification of ACS versus sCAD and ruptured versus stable plaques based on the THBS1+ macrophage gene signature. (A) Bar plot comparing AUC values for 13 classifiers trained on the GSE236610 dataset and evaluated on the GSE41571 testing set (SVM Linear and Naive Bayes, AUC = 0.9333). (B) ROC curve for the SVM Linear classifier (AUC = 0.9333). (C) ROC curve for the Naive Bayes classifier (AUC = 0.9333). (D) Box plot comparing SVM Linear risk scores between “risk” and “stable” groups in the testing set (two‐sided Wilcoxon rank‐sum test, *p* = 0.006). (E) Box plot comparing Naive Bayes risk scores between “risk” and “stable” groups (two‐sided Wilcoxon rank‐sum test, *p* = 0.015).

Model‐derived classification scores from SVM Linear were significantly higher in ruptured plaques than in stable plaques in the testing cohort (*p* = 0.006) (Figure 7D). Similarly, Naive Bayes‐derived classification scores were significantly higher in ruptured plaques than in stable plaques (*p* = 0.015) (Figure 7E). These findings suggest that the THBS1+ macrophage‐associated NEW10 module captures transcriptional features shared between ACS‐related clinical instability and morphological plaque rupture. However, the present results demonstrate cross‐sectional discriminatory performance rather than prediction of future plaque rupture, ACS, or major adverse cardiovascular events.

## 4 Discussion

In this study, we generated a high‐resolution single‐cell transcriptomic atlas of human coronary atherosclerotic plaques and characterized the phenotypic heterogeneity, developmental trajectories, gene regulatory architectures, and intercellular communication networks of plaque‐resident macrophages. Our analysis identified six transcriptionally distinct macrophage subpopulations, with the THBS1+ subset occupying a terminal position along the inferred pseudotemporal trajectory and exhibiting hypoxia‐related and TGF‐β‐enriched transcriptional features, together with putative FOSB‐associated regulatory activity inferred by pySCENIC. Notably, CellChat analysis predicted a THBS1‐CD36 interaction between THBS1+ macrophages and lymphatic endothelial cells, while computational docking and molecular dynamics simulations supported a predicted interaction between SMS121 and CD36. Furthermore, leveraging the THBS1+ macrophage‐specific transcriptional module NEW10, we developed machine learning classifiers that distinguished ACS from sCAD in the training cohort and showed discriminatory performance for ruptured versus stable plaques in an independent cohort. These findings suggest that the NEW10 signature captures transcriptional features associated with coronary atherosclerotic instability across related but non‐equivalent clinical and morphological contexts.

Our identification of six macrophage subpopulations aligns with emerging single‐cell studies in cardiovascular disease that have challenged the traditional M1/M2 dichotomy by revealing far greater phenotypic complexity (Ijaz et al. 2026; Yang et al. 2025). The PLD4+ and SPP1+ subsets displayed transcriptional features consistent with inflammatory and osteoclast‐like states, respectively, which have previously been implicated in plaque destabilization (Huang et al. 2024; Gavin et al. 2018), whereas the MX1+ subset may reflect an interferon‐primed antiviral state (Wang et al. 2024). The THBS1+ subpopulation, however, emerged as particularly distinctive. Its elevated hypoxia signature and TGF‐β pathway activity suggest that these cells reside in poorly oxygenated, fibrotic niches within the plaque—microenvironmental conditions known to promote collagen deposition and fibrous cap thickening, yet paradoxically also associated with advanced plaque maturation and vulnerability (Pervaiz et al. 2023). The effects of TGF‐β signaling in atherosclerosis are highly context dependent and vary according to the responding cell type and local microenvironment. Experimental inhibition of TGF‐β signaling has been shown to increase plaque inflammation and reduce collagen content, whereas macrophage‐specific TGF‐β1 overexpression reduces lesion burden and increases smooth muscle cell and collagen content, supporting a potential role in fibrous cap stabilization (Mallat et al. 2001; Reifenberg et al. 2012). In contrast, endothelial TGF‐β signaling can promote vascular inflammation, endothelial permeability, and atherosclerotic plaque progression (Chen et al. 2019). Moreover, TGF‐β can exert opposing effects on angiogenesis through distinct endothelial receptor pathways, with ALK1 signaling promoting endothelial cell migration and proliferation and ALK5 signaling suppressing these processes (Goumans et al. 2002). Therefore, TGF‐β pathway enrichment in THBS1+ macrophages should not be interpreted as intrinsically protective or deleterious, as its net effect likely depends on plaque stage, cellular target, and receptor context. Elevated FOSB regulon activity in this subset suggests a possible association with stress‐responsive transcriptional regulation, although direct regulation of THBS1 or other NEW10 genes by FOSB remains to be experimentally established (Robison and Nestler 2022). From a developmental perspective, pseudotemporal trajectory analysis positioned THBS1+ macrophages at a terminal branch point, which is consistent with a more differentiated transcriptional state but does not establish a definitive lineage relationship (Chelvanambi et al. 2024; An et al. 2025).

Cell–cell communication analysis further identified distinct predicted signaling patterns associated with THBS1+ macrophages. The marked attenuation of MIF signaling, coupled with the upregulation of THBS‐mediated communication, suggests a potential shift from pro‐inflammatory chemotaxis toward adhesive, matrix‐modulating interactions. Among these predictions was a putative THBS1‐CD36 interaction between THBS1+ macrophages and lymphatic endothelial cells. However, because CellChat infers ligand‐receptor communication from gene‐expression patterns, this prediction does not establish protein‐level binding, receptor activation, downstream signaling, or biological consequences. CD36 is a multifunctional scavenger receptor with well‐documented roles in lipid uptake, inflammation, and angiogenesis (Zhang et al. 2026). Arterial lymphatic vessels may exert protective effects by facilitating macrophage reverse cholesterol transport, immune‐cell egress, and lipid clearance from the arterial wall (Martel et al. 2013; Rademakers et al. 2017). However, lymphatic expansion has also been observed in advanced and ruptured plaques, and activated lymphatic endothelial cells can produce chemotactic signals that promote inflammatory‐cell recruitment, suggesting that their functions may vary with plaque stage and local inflammatory conditions (Rademakers et al. 2017; Kang et al. 2009). Within this context, the predicted THBS1‐CD36 interaction may influence lymphatic endothelial cell behavior, lipid drainage, or immune‐cell trafficking, but our computational analysis cannot determine whether this crosstalk promotes plaque progression or regression. Moreover, previous evidence indicates that THBS1 can regulate lymphatic endothelial cells through CD47, highlighting the need to experimentally confirm the receptor specificity of the predicted THBS1‐CD36 interaction (Singla et al. 2023). Future ligand‐receptor blockade experiments, lymphatic endothelial cell migration and tube‐formation assays, and in vivo studies will be required to define the functional consequences of this macrophage‐lymphatic endothelial cell crosstalk (Rademakers et al. 2017).

The computational prediction that SMS121 may interact with CD36 with favorable thermodynamics and conformational stability, as evidenced by molecular docking and 100‐ns molecular dynamics simulations, provides an initial structural rationale for pharmacological interception of this pathological axis (Fu et al. 2023; Guerrero‐Rodríguez et al. 2025). While *in silico* predictions require experimental validation through surface plasmon resonance, cellular uptake assays, and in vivo efficacy studies, the predicted binding affinity and trajectory stability of the SMS121‐CD36 complex warrant further experimental investigation (Fu et al. 2023; Guerrero‐Rodríguez et al. 2025). If validated, SMS121 could represent a first‐in‐class molecule capable of disrupting macrophage‐lymphatic crosstalk without globally abrogating CD36‐mediated lipid metabolism, thereby minimizing off‐target metabolic liabilities.

From a clinical standpoint, our machine learning analysis provides preliminary evidence that the NEW10 module may serve as a molecular signature of coronary atherosclerotic instability. Importantly, the two datasets used in this analysis captured different dimensions of disease instability. The GSE236610 training cohort was classified according to clinical presentation as ACS or sCAD, whereas the GSE41571 testing cohort was classified according to plaque morphology as ruptured or stable plaques. Although ACS and plaque rupture are biologically related, they are not synonymous, because ACS may occur through mechanisms other than overt plaque rupture and plaque rupture does not invariably result in an acute clinical event. The ability of the SVM Linear and Naive Bayes classifiers to achieve AUC values of 0.9333 in distinguishing ruptured from stable plaques therefore suggests that an ACS‐associated NEW10 signature may generalize to morphological plaque instability. However, the present analysis demonstrates cross‐sectional discrimination between related disease phenotypes rather than prospective prediction of plaque rupture, incident ACS, or major adverse cardiovascular events. Accordingly, the NEW10 score should currently be interpreted as a molecular classification score associated with coronary instability, rather than as a validated clinical risk score. Prospective studies with clearly adjudicated outcomes, including plaque rupture, recurrent ACS, and major adverse cardiovascular events, will be required to determine the specific clinical endpoint and incremental prognostic value of this signature.

Several limitations warrant acknowledgment. First, our analyses are constrained to publicly available scRNA‐seq datasets with relatively modest sample sizes, and the absence of paired clinical metadata precluded correlative analyses with patient outcomes. Second, CellChat infers ligand‐receptor interactions from transcript‐level expression and does not demonstrate protein–protein binding, receptor activation, downstream signaling, or biological function; therefore, the predicted THBS1‐CD36 interaction requires direct experimental validation. Likewise, the in silico nature of the SMS121‐CD36 interaction study necessitates experimental validation through in vitro binding assays and animal models of atherosclerosis. Third, the machine learning analysis used different outcome definitions in the training and testing cohorts. ACS versus sCAD represents a clinical classification, whereas ruptured versus stable plaques represents a morphological classification. Although external performance across these datasets supports the potential generalizability of the NEW10 signature, it does not establish that the model predicts a single predefined clinical endpoint. In addition, the small testing cohort of 5 ruptured and 6 stable plaques limits statistical power and may produce unstable performance estimates; therefore, the model should be considered preliminary. Future studies should validate it in larger, multicenter prospective cohorts with standardized endpoints and assess its added value beyond established clinical risk factors. Clinical application will also require a reproducible assay based on clinically accessible samples. Fourth, the regulatory role of FOSB was inferred computationally using pySCENIC, and whether FOSB directly binds to and regulates THBS1 or other genes within the NEW10 module remains to be determined. Future studies should combine FOSB knockdown or overexpression with ChIP‐qPCR or CUT&RUN, promoter‐reporter assays, and transcriptomic profiling to validate its direct targets and clarify its functional contribution to the THBS1+ macrophage phenotype. Finally, the spatial architecture of the coronary plaque—which likely influences macrophage‐lymphatic interactions—cannot be fully resolved by dissociated single‐cell approaches, suggesting that future spatial transcriptomic studies would complement our findings. Such studies are technically feasible using archived or prospectively collected plaque tissues and could validate the spatial colocalization of THBS1+ macrophages and lymphatic endothelial cells, thereby providing stronger evidence for the predicted intercellular interaction.

In conclusion, our integrative single‐cell and computational framework characterizes macrophage heterogeneity, associated transcriptional states, and predicted intercellular communication patterns in coronary atherosclerosis, identifies the predicted THBS1‐CD36 interaction as a candidate mechanism requiring experimental validation, and defines the NEW10 module as a candidate molecular signature associated with coronary atherosclerotic instability across clinical and morphological contexts. Further prospective studies are required to establish whether this signature predicts plaque rupture, ACS, major adverse cardiovascular events, or another clinically relevant endpoint. These findings provide a basis for further investigation of THBS1+ macrophages and their associated molecular features in coronary atherosclerosis.

## 5 Conclusions

In this study, we identified six transcriptionally distinct macrophage subpopulations in coronary atherosclerotic plaques and characterized the THBS1+ subset by a terminally differentiated, hypoxia‐responsive, and TGF‐β‐enriched transcriptional state. CellChat analysis predicted a putative THBS1‐CD36 interaction between THBS1+ macrophages and lymphatic endothelial cells, while molecular docking and molecular dynamics simulations provided preliminary computational support for an interaction between SMS121 and CD36 that requires experimental validation. The THBS1+ macrophage‐associated NEW10 signature also showed discriminatory performance across clinical and morphological manifestations of coronary instability, although validation in larger prospective cohorts is needed. These findings provide a framework for further mechanistic, pharmacological, and translational investigation of macrophage heterogeneity in coronary atherosclerosis.

## Author Contributions

**Jianpeng Li:** conceptualization, writing – original draft, data curation, formal analysis. **Xinrui Liu:** data curation, formal analysis. **Run Shi:** formal analysis. **Lin Yang:** supervision. **Rong Gu:** supervision, writing – review and editing.

## Funding

The authors have nothing to report.

## Ethics Statement

This study is based on published or public datasets and does not require ethical approval and consent.

## Consent

All authors have read and approved the final manuscript.

## Conflicts of Interest

The authors declare no conflicts of interest.

## Data Availability

The data that support the findings of this study are available from the corresponding author upon reasonable request.
